# Routine anaesthesia ward-based patient visits in surgery: 1-year outcomes of the TRACE randomized clinical trial

**DOI:** 10.1093/bjs/znaf019

**Published:** 2025-03-12

**Authors:** Valérie M Smit-Fun, Dianne de Korte-de Boer, Thomas Damen, Annick Stolze, Linda M Posthuma, Markus W Hollmann, Wolfgang F F A Buhre, the TRACE Study Investigators Group, C Boer, S van Kuijk, P G Noordzij, M Rinia, J P Hering, B in ’t Veld, G J Scheffer, J S Breel, T Bouw, F van Dijk, J Geurts, W Glas, R van Gorp, A Jwair, F Koca, I Lange, B Preckel, J P van Roy, M Theunissen, A G C L Wensing, A Werger

**Affiliations:** Department of Anaesthesia and Pain Medicine, Maastricht University Medical Centre +, Maastricht, The Netherlands; Department of Anaesthesia and Pain Medicine, Maastricht University Medical Centre +, Maastricht, The Netherlands; Department of Anaesthesia and Pain Medicine, Maastricht University Medical Centre +, Maastricht, The Netherlands; Department of Anaesthesia, Amsterdam University Medical Centre, Amsterdam, The Netherlands; Department of Anaesthesia and Intensive Care, Albert Schweitzer Hospital, Dordrecht, The Netherlands; Department of Anaesthesia, Amsterdam University Medical Centre, Amsterdam, The Netherlands; Department of Anaesthesia, University Medical Centre Utrecht, Utrecht, The Netherlands

## Abstract

**Background:**

The TRACE (Routine posTsuRgical Anaesthesia visit to improve patient outComE) RCT did not show any perioperative benefit from ward-based visits by anaesthetists after surgery. The aim of this study was to evaluate the impact of this intervention on longer-term outcomes.

**Methods:**

Patients were followed up in the TRACE RCT to 1 year in nine hospitals in the Netherlands. Patients undergoing elective non-cardiac surgery, and at risk for adverse postoperative outcome, were included. Patients in the intervention group additionally received routine anaesthesia visits on postoperative days 1 and 3. Clinical outcome measures included 1-year mortality, hospital readmission, and reoperation. Functional recovery (FR) was measured using the patient-reported global surgical recovery (GSR) index, ability to perform activities of daily living (ADL), and functional recovery index (FRI). Quality of life (QoL) was measured using EQ-5D-5L.

**Results:**

Some 5473 adult patients were followed up. No differences were found between the control and intervention groups for clinical, FR, and QoL outcome measures. One-year mortality was 5.4% in the control group and 5.8% in the intervention group, readmission was 27% and 26% respectively, and reoperation was 20% and 18% respectively. At 1 year, FR and QoL had recovered to preoperative levels. However, 30% of patients were not able to fully perform ADL and 40%–51% of patients still reported a problem in the EQ-5D-5L dimensions mobility, usual activities, and pain/discomfort.

**Conclusion:**

Routine postoperative anaesthesia ward visits of patients did not improve clinical, functional, and QoL outcomes. A substantial proportion of patients still experienced health-related limitations in daily life 1 year after surgery. In conclusion, an early postoperative intervention with postoperative anaesthesia visits in the ward after non-cardiac surgery had no effect on 30-day or 1-year clinical outcome. Remarkably, TRACE shows that compared with data sampled 10 years ago, 1-year mortality has not improved in the Netherlands. At 1 year, functional recovery or QoL showed little improvement compared with baseline. Importantly, a substantial number of patients still reported incomplete recovery and problems that limit QoL, which indicate that there is still room for improvement.

## Teaser

One-year evaluation showed that routine postoperative anaesthesia visits did not have an impact on outcome regarding clinical outcome measures, functional recovery, or quality of life. However, important lessons learned are that, although 30-day mortality was drastically reduced in the past decade, 1-year mortality remained the same, suggesting non-surgical determinants of clinical outcome that need to be considered before surgery. Furthermore, after 1 year, a considerable percentage of patients have not fully recovered and still experience limitations in their activities of daily living and in studied dimensions of quality of life, suggesting much room for intensified and prolonged postoperative care.

## Introduction

In perioperative medicine, prospective long-term studies on clinical and functional outcome after major surgery and in general surgical populations are scarce. Traditionally, clinical outcome measures such as postoperative complications, mortality, readmission, and reoperation are used to describe surgical patient outcome. More recently, studies increasingly focus on patient-centred outcome, such as patient-reported quality of life (QoL)^1–3^. Outcome after surgery is mostly studied in specific surgical patient populations, limiting generalizability to the general surgical population, and the follow-up intervals are often short^4,5^.

The authors performed the TRACE (Routine posTsuRgical Anaesthesia visit to improve patient outComE) study for which patients were randomly assigned to a control arm of routine care or an intervention group where patients were routinely visited after surgery on the ward by an anaesthetist^4^. The main outcome of the TRACE study was postoperative complications and mortality until 30 days after surgery. This has been published previously and demonstrated no difference between control and intervention arms, with an overall low 30-day mortality rate of 0.5% and a low incidence of failure to rescue^5^. The aim of this study was to evaluate the impact of postoperative ward visits by anaesthetists on patient outcomes during 1-year follow-up of the TRACE RCT.

## Methods

### Study design

A full description of the TRACE study was previously published^4^. Briefly, TRACE is a prospective, multicentre, stepped-wedge, cluster-randomized interventional study in patients who underwent non-cardiac surgery in nine academic and non-academic hospitals in the Netherlands. According to the stepped-wedge randomization, eligible patients were enrolled in either the control group or the intervention group. Patients in the control group received postoperative care as usual provided by the surgical ward team (nurses and physicians) and included daily visits by the ward team and retrieval of the Modified Early Warning Score (MEWS) three times a day. Patients in the intervention group received additional routine postoperative anaesthesia visits on days 1 and 3. Their clinical condition was evaluated with the aim to timely recognize patient deterioration and avoid complications and failure to rescue. Based on the anaesthetist’s findings recommendations were given for adjustments of care. Data on the number and nature of recommendations provided were previously published^5^.

Ethical approval was obtained from the Human Subjects Committee of Amsterdam UMC, location VUmc Amsterdam (NL56004.029.16). The study was registered in the Netherlands Trial Register (NTR5506). Patient inclusion and data registration was monitored by the Clinical Research Unit of the Amsterdam UMC.

### Participants

Study participants were in-hospital non-cardiac surgical patients, who consented to the study and met one or more of the following criteria: age ≥60 years; age ≥45 years with a revised cardiac risk index (rCRI) >2; age ≥18 years with an indication for postoperative invasive pain therapy; and/or age ≥18 years with a postoperative Surgical Apgar Score (SAS) score <5. Patients with an indication for a postoperative ICU stay were excluded.

### Outcome measures

TRACE is a study with a longitudinal follow-up until 1 year after surgery. Previously, short-term clinical outcome and QoL measures (for example mortality, complications, and EQ-5D-5L index) until 30 days were studied and reported^5^. For the present paper, clinical, functional recovery (FR), and QoL outcome measures were studied until 1 year. Although previously studied and reported^5^, relevant data from time points until 30 days after surgery were included in this 1-year study to present the full course of outcome measures from baseline until 1 year. Based on the absence of outcome differences between study groups at 30 days, earlier intended economic evaluation of the intervention at 1 year, as proposed in the trial protocol^4^, is not taken into account, as no cost-effectiveness benefit is expected. Descriptions and definitions of presented study outcome measures are given in the *Supplementary material*.

Data on patient survival until 1 year after surgery were collected from the hospital patient records and from the National Register of the Deceased. Data on hospital readmission, reoperations, FR, and QoL were collected from patient questionnaires at baseline and day 7, day 30, and 1 year after surgery.

To assess FR, three self-reported instruments were employed: the global surgical recovery (GSR) index^6^; the ability to perform activities of daily living (ADL); and the functional recovery index (FRI)^7^.

To assess QoL, the study was registered at EuroQol and the EQ-5D-5L questionnaire, including a visual analogue scale (EQ-5D-5L VAS), from which the EQ-5D-5L index score was calculated using the value set for the Dutch population, was used^8^.

### Statistical analysis

Patient characteristics are described using mean(s.d.) or median (interquartile range (i.q.r.)) for continuous variables, depending on the distribution of the variable, and *n* (%) for categorical variables. The 1-year mortality was compared between groups using logistic mixed-effects regression analysis with a random intercept for hospital. Group differences were adjusted for time effects and baseline characteristics that differed between groups to a clinically meaningful extent. Given the study design, additional correction for type 1 error was not necessary. Secondary outcomes were tested between groups using linear or logistic mixed-effects regression, or non-parametric tests, depending on the distribution of the outcome, with a similar random-effects structure as for the primary outcome measure. Analyses were performed according to the intention-to-treat principle. All analyses were performed using SPSS^®^ (IBM, Armonk, NY, USA; Statistics for Windows, version 28).

### Patient and public involvement

Patient partners were not involved in the design and conduct of this study. Before initiation of the study, the study proposal was reviewed by the Dutch Patient and Consumers Federation (NPCF). They considered the study relevant and therefore supported the study. Additionally, the study was discussed with patients in collaboration with the Dutch Anaesthesia Society.

## Results

### Study population

**Fig. 1:**
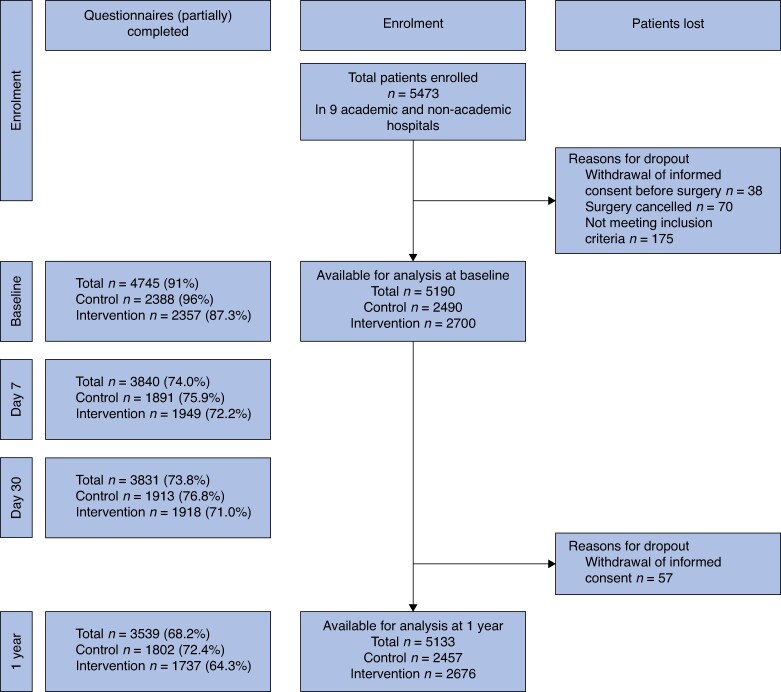
Patient flow diagram

A total of 5473 patients were included in the study. Of these, 5190 patients (2700 in the intervention group and 2490 in the control group) were eligible for analyses at 30 days after surgery. Between 30 days and 1 year after surgery, 57 patients withdrew informed consent, resulting in 5133 patients (2457 in the control group and 2676 in the intervention group) eligible for 1-year follow-up (*Fig. 1*). At baseline, day 7, day 30, and 1 year, 91.4%, 74.0%, 73.8%, and 68.2% of all questionnaires were (partially) completed (*Fig. 1*). Baseline patient and surgical data are presented in *Table 1*.

**Table 1:** Baseline characteristics

	Control group (*n* = 2490)	Intervention group (*n* = 2700)	Total (*n* = 5190)
Age (years), median (i.q.r.)	67 (61–73)	67 (61–73)	67 (61–73)
**Sex**			
Female	1169 (47.0)	1300 (48.1)	2469 (47.6)
BMI (kg/m^2^), median (i.q.r.)	27 (24–31)	27 (24–31)	27 (24–31)
Activity level (MET score <4)	151 (6.1)	155 (5.7)	306 (5.9)
**Functional status**			
Independent	2278 (91.5)	2484 (92.0)	4762 (91.8)
Partially independent	173 (7.0)	209 (7.7)	382 (7.4)
Totally dependent	9 (0.4)	7 (0.3)	16 (0.3)
Unknown	30 (1.2)	0 (0.0)	30 (0.6)
**ASA classification**			
I	231 (9.3)	232 (8.6)	463 (8.9)
II	1536 (61.7)	1627 (60.3)	3163 (60.9)
III	697 (28.0)	803 (29.7)	1500 (28.9)
IV	23 (0.9)	37 (1.4)	60 (1.2)
Unknown	3 (0.1)	1 (0.0)	4 (0.1)
**Co-morbid disorders**			
Active cancer	945 (38.0)	1153 (42.7)	2098 (40.4)
Hypertension	1131 (45.4)	1187 (44.0)	2318 (44.7)
Cardiovascular disease	654 (26.3)	706 (26.1)	1360 (26.2)
Cerebrovascular disease	181 (7.3)	196 (7.3)	377 (7.3)
Diabetes mellitus	381 (15.3)	411 (15.2)	792 (15.3)
Pulmonary disease	256 (10.3)	283 (10.5)	539 (10.4)
Renal failure	190 (7.6)	351 (13.0)	541 (10.4)
Type of surgery*			
Ear, nose, and throat	76 (3.1)	96 (3.6)	172 (3.3)
Gastrointestinal or liver	749 (30.1)	902 (33.4)	1651 (31.8)
Gynaecological	204 (8.2)	204 (7.6)	408 (7.9)
Orthopaedic, arthroplasty, and spine	479 (19.2)	473 (17.5)	952 (18.3)
Thoracic	108 (4.3)	117 (4.3)	225 (4.3)
Urological	442 (17.8)	457 (16.9)	899 (17.3)
Vascular	168 (6.7)	207 (7.7)	375 (7.2)
Other	348 (14.0)	283 (10.5)	631 (12.2)
**Grade of surgery†**			
High-risk surgery	1085 (43.6)	1105 (40.9)	2190 (42.2)

Values are *n* (%) unless otherwise indicated. *Some patients may have had more than one type of surgery. †According to the revised cardiac risk index (intraperitoneal, intrathoracic, or suprainguinal vascular surgery). i.q.r., interquartile range; MET, metabolic equivalent of task.

### Anaesthesia recommendations

Postoperative recommendations were given for 437 patients (16.2%) on day 1 and 293 patients (10.9%) on day 3. The subgroup of patients for which any advice was given on day 1 or 3 consists of 582 patients (21.6%). The majority of recommendations consisted of optimization of pain therapy (28% of the provided advice on day 1 and 23% on day 3), adjustment of medication (15% and 16%), or adherence to elements of the enhanced recovery after surgery guidelines (40% and 42%). Of all, recommendations were followed in 67% of cases on day 1 and in 69% on day 3.

### Mortality

At 1 year after surgery, 289 patients (5.6%) had died—133 patients (5.4%) in the control group and 156 patients (5.8%) in the intervention group (adjusted OR 0.98 (95% c.i. 0.74 to 1.31), *P* = 0.897) (*Table S1*). Of the 289 deceased patients, 221 (76.5%) were known to have cancer at baseline; this proportion was not significantly different between groups (73.7% in the control group and 78.8% in the intervention group; *P* = 0.302).

### Readmission and reoperation

Within 1 year after surgery, 450 patients (26.6%) in the control group and 413 patients (25.7%) in the intervention group were readmitted to the hospital (adjusted OR 0.91 (95% c.i. 0.75 to 1.10), *P* = 0.305). In the control group, 347 patients (20.5%) required reoperation, whereas 292 patients (18.1%) in the intervention group were reoperated on within 1 year after surgery (adjusted OR 0.83 (95% c.i. 0.68 to 1.02), *P* = 0.074) (*Table S1*). The overall readmission rate was 26.2% and the overall reoperation rate was 19.3%.

### FR

#### GSR index

The median GSR index increased over time without a difference between groups. At postoperative day 7 the median GSR index was 60% in both groups (*P* = 0.941), at 30 days it was 75% in both groups (*P* = 0.189), and at 1 year it was 90% in the control group and 95% in the intervention group (*P* = 0.327) (*Table S2* and *Fig. S1*). Before surgery, the patients’ median expected GSR index was 60% in the control group and 50% in the intervention group for day 7 after surgery (*P* = 0.551) and 85% in the control and 90% in the intervention group for day 30 after surgery (*P* = 0.293) (*Table S2*).

#### ADL

The proportion of patients feeling capable of completely performing their daily activities was 16.6% in the control group and 13.7% in the intervention group (adjusted OR 0.74 (c.i. 0.60 to 0.92), *P* = 0.007) at day 7 after surgery, 34.8% and 32.9% respectively (adjusted OR 0.91 (c.i. 0.76 to 1.09), *P* = 0.302) at day 30 after surgery, and 68.4% and 69.8% respectively (adjusted OR 1.07 (c.i. 0.89 to 1.30), *P* = 0.466) at 1 year after surgery (*Table S3* and *Fig. S1*).

#### FRI

*Table S4a*,*b* and *Fig. S2* display FRI scores from baseline until 1 year after surgery per study group. The table includes overall FRI scores, scores per domain, and the percentage of patients with a score ≥1 (experiencing at least some difficulty in the given domain). The latter is also displayed in *Fig. S3*. Until 1 year, the overall median FRI scores were 16 in the control group and 18 in the intervention group at baseline (*P* = 0.882), 64 and 68 respectively at day 7 (*P* = 0.299), 33 in both groups at day 30 (*P* = 0.684), and 11 in both groups at 1 year (*P* = 0.649). There were no significant differences between groups at any time point. Until 1 year, the proportion of patients having at least some problem showed the same pattern over time, with worse scores at day 7 and with 1-year scores comparable to baseline. Remarkably, at 1 year at least 69.0% of patients reported a functional limitation.

### QoL

#### EQ-5D-5L index

The median EQ-5D-5L index score for the control group and the intervention group was 0.82 (i.q.r. 0.62–0.91) *versus* 0.82 (i.q.r. 0.66–0.91) at baseline, 0.74 (i.q.r. 0.58–0.85) *versus* 0.74 (i.q.r. 0.60–0.85) at day 7, 0.81 (i.q.r. 0.70–0.89) *versus* 0.81 (i.q.r. 0.70–0.89) at day 30, and 0.86 (i.q.r. 0.74–1.00) *versus* 0.88 (i.q.r. 0.75–1.00) at 1 year. None of these scores differed significantly between groups (*Table S5*).

#### EQ-5D-5L VAS

**Fig. 2:**
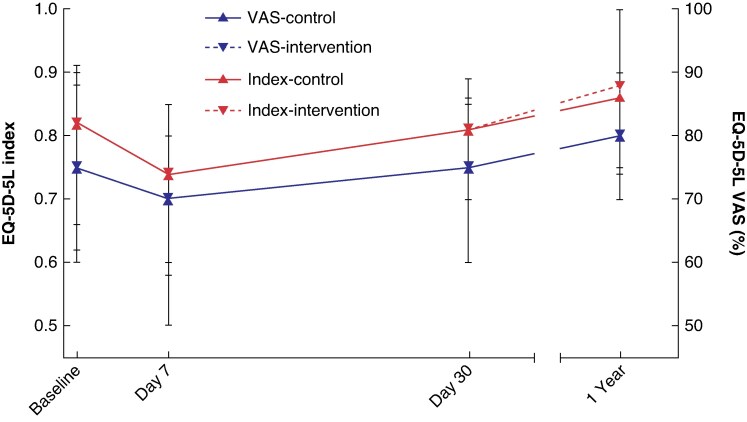
EQ-5D-5L VAS and EQ-5D-5L index for the control and intervention groups VAS, visual analogue scale.

The median EQ-5D-5L VAS for the control group and the intervention group was 75 (i.q.r. 60–88) *versus* 75 (i.q.r. 60–90) at baseline, 70 (i.q.r. 50–80) *versus* 70 (i.q.r. 50–80) at day 7, 75 (i.q.r. 60–85) *versus* 75 (i.q.r. 60–86) at day 30, and 80 (i.q.r. 70–90) *versus* 80 (i.q.r. 70–90) at 1 year (*Fig. 2*). Overall, QoL was not significantly different between the control group and the intervention group at any time point. The EQ-5D-5L index and EQ-5D-5L VAS followed the same pattern (*Fig. 2*), suggesting internal consistency.

#### EQ-5D-5L dimensions

**Fig. 3:**
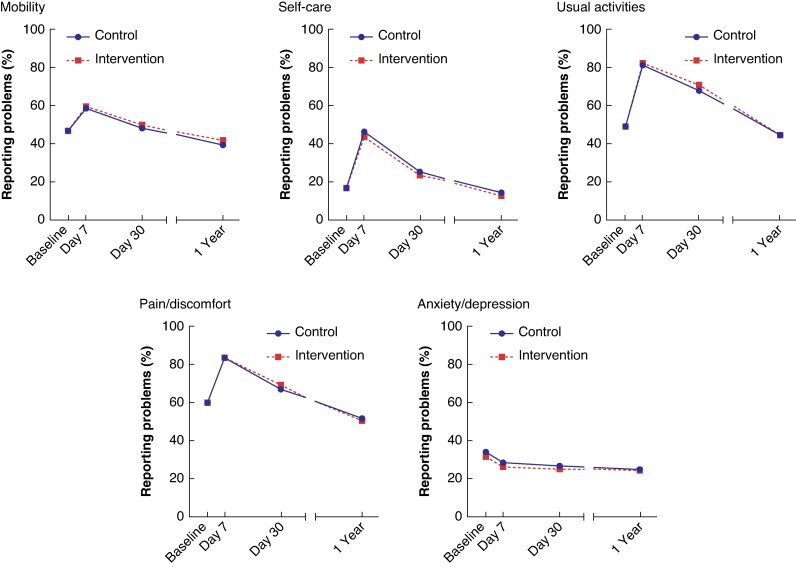
Percentage of patients reporting a problem within EQ-5D-5L dimensions from baseline up to 1 year after surgery Reporting a problem was defined as an EQ-5D-5L dimension response of ≥2.

*Fig. 3* and *Table S6* display the proportion of patients reporting a problem in the EQ-5D-5L dimensions (mobility, self-care, usual activities, pain/discomfort, and anxiety/depression) at several time points from baseline until 1 year after surgery. In all dimensions, except for anxiety/depression, this proportion is highest at day 7. Compared with baseline, patients scored better at 1 year in all dimensions, but still 40% of patients experienced problems with mobility, 14% with self-care, 45% with usual activities, 51% with pain/discomfort, and 24% with anxiety/depression.

### Post-hoc subgroup analyses of patients in the intervention group with recommendations

In post-hoc analyses within the intervention group, outcome measures were compared between the subgroup of patients for which any advice was given on day 1 or 3 (582 patients (21.6%)) and the subgroup for which no recommendations were given (2118 patients (78.4%)). At baseline, patients with recommendations were slightly older, more likely to be female, more likely to have a metabolic equivalent of task (MET) score <4, less likely to be functionally independent, more likely to have hypertension, cardiovascular disease, and diabetes, and more likely to have undergone high-risk surgery (*Table S7*). The incidence of mortality after 1 year was higher in the subgroup of patients for whom a recommendation was given: 43 (7.5%) died compared with 113 (5.4%) of patients in the intervention group for whom no recommendation was given. Furthermore, a higher proportion of patients for whom a recommendation was given was readmitted to the hospital within 30 days and more reoperations occurred in this group of patients. With respect to patient-reported outcomes, the subgroup of patients with recommendations had poorer outcomes regarding the GSR index on day 7, day 30, and 1 year, and a lower proportion of patients in this subgroup reported being able to perform ADL on day 7, day 30, and 1 year. In addition, patients for whom recommendations were given had higher median FRI sum scores on day 7, day 30, and 1 year, indicating less functional independence, and lower median EQ-5D-5L index scores on day 7, day 30, and 1 year, indicating lower QoL (*Table S8*).

## Discussion

The TRACE study evaluated the effect of postoperative anaesthesia visits on clinical outcome, FR, and QoL in patients undergoing elective non-cardiac surgery. At 1 year after surgery, no significant effect of the anaesthesia visit was detected on these outcome measures.

The 1-year postsurgical mortality (overall 5.6%) is on the low end of reported mortality rates from other studies in high-income countries (4.8–17.6%)^9–12^ and is similar to Dutch mortality data presented in 2012^13^. This is striking, because 30-day mortality in TRACE was substantially lower than previously reported (TRACE 0.5% *versus* 2% in 2012)^5,13–15^. Independent of a low 30-day mortality, long-term postsurgical mortality has not improved within the last ten years in the Netherlands. Probably, the main factors contributing to 1-year postsurgical mortality are patient and disease related, and not (or less so) surgery related^16–20^.

The 1-year readmission rates are comparable to earlier data^21^. In the present study, the reported reoperation rate (overall rate of 19.3%) is comparable to the average reported rates in specific surgical patient cohorts (7.4–35.4%)^22–27^. Data from general surgical populations are not available. Approximately 66% of readmissions occur within 30 days after surgery^28,29^. Thereafter, the direct relation between readmission and index surgery decreases^29,30^. It is therefore likely that the majority of readmissions and reoperations in the TRACE population between 30 days and 1 year are not directly related to the index operation.

TRACE presents several dimensions of FR, whereas most previous studies focused on recovery of physical functioning, which is only part of what patients consider important in their recovery^30–32^. For QoL, the dimensions are also presented besides the EQ-5D-5L index.

Both FR and QoL show similar patterns of decline and recovery, but with different timelines. At 1 week after surgery, patients showed severe functional decline, with patients reporting to feel approximately 60% recovered according to the GSR index and only 15% of patients were able to fully perform their ADL. Looking at the expected GSR index, the FR was considerably lower than patients’ preoperative level of expectation for day 30 after surgery. The FRI was only comparable to baseline at 1 year after surgery. The findings of the present study, that approximately 30% of patients report functional limitations in at least one FRI dimension at 1 year, with approximately 30% of patients not being able to fully perform ADL, correspond with findings of previous studies^30,32^.

In line with FR, QoL showed limited improvement. At 30 days, EQ-5D-5L VAS and the EQ-5D-5L index showed a return to at least preoperative values. At 1 year, the difference between the median EQ-5D-5L index scores at 1 year and baseline was 0.04 and 0.06 for the control group and the intervention group respectively. This is lower than the minimally important difference (MID) of 0.08 in comparable European countries^33^ and thus suggests no overall benefit of surgery on QoL at group level. In line, a substantial percentage of patients still reports problems in their QoL, and EQ-5D-5L VAS suggests that patients had only improved to three-quarters of their best imaginable health state at 1 year.

In view of FR and QoL, TRACE shows that patients perceive a faster recovery of QoL than of FR and that surgery often does not result in full FR or full recovery of QoL. The reported limited recovery seems contrary to patients’ and surgeons’ expectations. This discrepancy could be explained by patients’ preoperative expectancy of complete recovery and the resulting postoperative health status of which the latter is more concordant with surgeons’ expectations^34,35^. This calls for surgeons and anaesthetists to invest in fully understanding patients’ needs and expectations and in methods to improve preoperative counselling^36,37^. Further studies are needed to determine which patients are prone to incomplete recovery, what the limiting factors are, whether further improvement is feasible, and what measures are needed to further improve recovery in this population. Potential interventions could be close monitoring and intensified patient follow-up after hospital discharge, prolonged pain monitoring and management, and interventions like extended physiotherapy and nutritional support in the interval after surgery. The suggested study information could aid in better preoperative patient information and expectations, decision support regarding the choice of treatment, and so in better QoL and use of healthcare resources.

The EQ-5D-5L dimension anxiety/depression shows a different time pattern, with a peak in reported problems before surgery instead of after surgery. This is most likely related to preoperative surgical anxiety. It shows that this measurement is sensitive to a life event such as an imminent surgical procedure, leading to underestimation of QoL at baseline.

The results of the post-hoc subgroup analyses show that the subgroup of patients for whom recommendations were given involved patients with poorer preoperative status and poorer postoperative recovery compared with patients in the intervention group without recommendations. Likely, the anaesthetists performing the visits had appropriately identified a more vulnerable subpopulation that was at increased risk of poorer surgical outcome and adequately foresaw the need for closer monitoring, follow-up, and intensified (collaborative) postoperative care. Whether or not more strict following of recommendations would have resulted in better postoperative outcomes remains unknown.

The data from TRACE on FR and return of QoL could be used for patient education and setting expectations, but is also important to direct future research towards improving value of care. The authors therefore recommend including these data in future studies.

TRACE provided outcome data over the first 30 days and at 1 year after surgery, but not at the intermediate time point (3–6 months), limiting the understanding of the time between 30 days and 1 year, and possibly allowing recall bias in the self-reported evaluation at 1 year. Specifically, information on readmission and reoperation until 1 year after surgery was only patient reported and could not always be verified by the researchers. As could be expected in prospective studies with a prolonged follow-up, there was a substantial rate of incomplete questionnaire data (31.8%) at 1 year. Although slightly more questionnaire data were missing in the intervention group compared with the control group after 1 year, the authors see no reason for a systematic cause of missingness and therefore assume no bias was introduced in the comparison between the study arms. However, generalizability of results may be hampered, because typically loss to follow-up occurs in the very sick, who are not able to complete the questionnaire, and in the relatively healthy participants, who have other priorities, for example because they have returned to work.

## Collaborators

The TRACE Study Investigators Group: C. Boer (Amsterdam University Medical Centre, Amsterdam, The Netherlands); S. van Kuijk (Maastricht University Medical Centre +, Maastricht, The Netherlands, Maastricht University, Maastricht, The Netherlands); P. G. Noordzij (St. Antonius Hospital, Nieuwegein, The Netherlands); M. Rinia (Rijnstate Hospital, Arnhem, The Netherlands); J. P. Hering (Dijklander Hospital, Hoorn, The Netherlands); B. in ’t Veld (Medical Centre Haaglanden, The Hague, The Netherlands); G. J. Scheffer (Radboud University Medical Centre, Nijmegen, The Netherlands); J. S. Breel (Amsterdam University Medical Centre, Amsterdam, The Netherlands); T. Bouw (Maastricht University Medical Centre +, Maastricht, The Netherlands); F. van Dijk (Rijnstate Hospital, Arnhem, The Netherlands); J. Geurts (Rijnstate Hospital, Arnhem, The Netherlands); W. Glas (Dijklander Hospital, Hoorn, The Netherlands); R van Gorp (Maastricht University Medical Centre +, Maastricht, The Netherlands); A. Jwair (Amsterdam University Medical Centre, Amsterdam, The Netherlands); F. Koca (Amsterdam University Medical Centre, Amsterdam, The Netherlands); I. Lange (St. Antonius Hospital, Nieuwegein, The Netherlands); B. Preckel (Amsterdam University Medical Centre, Amsterdam, The Netherlands); J. P. van Roy (Dijklander Hospital, Hoorn, The Netherlands); M. Theunissen (Maastricht University Medical Centre +, Maastricht, The Netherlands); A. G. C. L. Wensing (Amsterdam University Medical Centre, Amsterdam, The Netherlands); A Werger (Medical Centre Haaglanden, The Hague, The Netherlands)

## Supplementary Material

znaf019_Supplementary_Data

## Data Availability

Data collected for the study, including deidentified individual participant data that underlie the results reported in this article (text, tables, and figures), to achieve the aims in the approved proposal will be made available to researchers who provide a methodologically sound proposal to the corresponding author with a signed data access agreement, beginning 3 months and ending 5 years following article publication.
