# Supplementary material for: Routine anaesthesia ward-based patient visits in surgery: 1-year outcomes of the TRACE randomized clinical trial
Source: Br J Surg. 2025 Mar 12;112(3):znaf019. doi: 10.1093/bjs/znaf019 (PMC11897594; doi:10.1093/bjs/znaf019)
Supplement: znaf019_Supplementary_Data [file znaf019_supplementary_data.docx]

**Title**

**Routine anaesthesia ward-based patient visits in surgery: 1-year outcomes of the TRACE randomized clinical trial**

**Authors**

Valérie M. Smit-Fun^a^, Dianne de Korte-de Boer^a^, Thomas Damen^a^, Annick Stolze^c^, Linda M. Posthuma^d^, Markus W. Hollmann^c^, Wolfgang F.F.A. Buhre^b^, On behalf of The TRACE Study Investigators Group

^a^ Department of Anaesthesia and Pain Medicine, Maastricht University Medical Centre +, Maastricht, The Netherlands

^b^ Department of Anaesthesia, University Medical Centre Utrecht, Utrecht, The Netherlands

^c^ Department of Anaesthesia, Amsterdam University Medical Centre, Amsterdam, The Netherlands

^d^ Department of Anaesthesia and Intensive Care, Albert Schweitzer Hospital, Dordrecht, The Netherlands

**Corresponding author. Name and address ORCID ID.**

V. Smit-Fun, Department of Anaesthesia and Pain Medicine, Maastricht University Medical Centre +, P.O. Box 5800, 6202 AZ Maastricht, The Netherlands, [v.smit.fun@mumc.nl](mailto:v.smit.fun@mumc.nl)

ORCID ID: 0000-0001-5528-853X

Supplementary Materials – Index

[Supplementary Appendixes](#_Supplementary_Appendixes)

[Abbreviations 3](#_Toc190965990)

[Definitions 3](#_Toc190965991)

[Overview of scores used in this study 5](#_Toc190965992)

[Revised Cardiac Risk Index (rCRI) 5](#_Toc190965993)

[Global Surgical Recovery (GSR) index 5](#_Toc190965994)

[Self-reported ADL recovery (ADL) 5](#_Toc190965995)

[Functional Recovery Index (FRI) 6](#_Toc190965996)

[EQ-5D-5L 7](#_Toc190965997)

[EQ-5D-5L VAS 8](#_Toc190965998)

[EQ-5D-5L (index) value 8](#_Toc190965999)

[Table S1. Adverse events until one year after surgery 9](#_Toc190966000)

[Table S2 Global Surgical Recovery Index 10](#_Toc190966001)

[Figure S1. Global Surgical Recovery and ADL performance 11](#_Toc190966002)

[Table S3. Ability to fully perform Activities of Daily Living (ADL) 12](#_Toc190966003)

[Table S4a. Functional Recovery Index 13](#_Toc190966004)

[Figure S2. Functional Recovery Index 15](#_Toc190966005)

[Figure S3. Functional Recovery Index overall and split per domain. 16](#_Toc190966006)

[Table S5: EQ-5D-5L index score 17](#_Toc190966007)

[Table S6. Distribution of TRACE EQ-5D-5L dimension responses at baseline and at postoperative follow-up. 18](#_Toc190966008)

[Table S7. Results of post-hoc subgroup analyses – baseline characteristics 19](#_Toc190966009)

[Table S8 Results of post-hoc subgroup analyses – outcome variables 20](#_Toc190966010)

[References](#_References)

# Supplementary Appendixes

## Abbreviations

**TRACE** Routine pos**T**su**R**gical **A**nesthesia visit to improve patient out**C**om**E** [1–3]

**rCRI** Revised Cardiac Risk Index [4]

**FR** Functional Recovery

**QoL** Quality of Life

**ADL** Activities of Daily Living

## Definitions

| **Intermediate-risk and high-risk for surgery patient** | Risk assessment was done according to the rCRI classification using and scoring 6 independent predictors as shown below:   \| **Clinical variable** \| **Points** \| \| --- \| --- \| \| High-risk surgery^a^ \| 1 \| \| Ischemic heart disease \| 1 \| \| Congestive heart failure \| 1 \| \| Cerebrovascular disease \| 1 \| \| Insulin treatment for diabetes mellitus \| 1 \| \| Pre-operative serum creatinine level > 2.0 mg/dl (180 mcg/mol/L \| 1 \|   The risk is interpreted based on the total score:   \| Risk class \| Points \| Risk of complications (%) \| \| --- \| --- \| --- \| \| 1. Very low \| 0 \| 0.4% \| \| 1. Low \| 1 \| 0.9% \| \| 1. Intermediate \| 2 \| 7.0% \| \| 1. High \| 3+ \| 11.0% \|   A patient in the TRACE study that is at intermediate or high risk is classified respectively in risk class III or IV.  ^a^ High-risk surgeries: intraperitoneal, intrathoracic or suprainguinal vascular surgery. |
| --- | --- | --- | --- | --- | --- | --- | --- | --- | --- | --- | --- | --- | --- | --- | --- | --- | --- | --- | --- | --- | --- | --- | --- | --- | --- | --- | --- | --- | --- | --- |
| **Readmission** | When a surgical patient has been discharged from the index hospital admission for a specific surgery, and is admitted again within 1 year after hospital discharge, regardless of the reason for the readmission. |
| **Re-operation** | An operation to correct a condition not corrected by the index operation or to correct the complications of the index operation after discharge from the index hospital admission, and within the 1-year follow-up period of the TRACE study. |
| **30-Day mortality** | Death from all causes during the hospitalisation for, or within 30 days of the index surgical procedure. |
| **1-Year mortality** | Death from all causes within 1 year of the index surgical procedure. |
| **Functional recovery** | The patient’s self-reported return to full health after surgery and anaesthesia, defined by the patient’s perception of general and psychological well-being and nociceptive burden, the return of physical independence (the ability to take care of one’s self), and the return of one’s physical, functional and cognitive capacity over time with comparison to baseline values prior to surgery.  To measure the FR in the TRACE study the following scores were used: the GSR index, ADL and the FRI. |
| **Quality-of-Life** | The patient’s self-reported health status in accordance to the EuroQoL defined domains mobility, self-care, daily activities, pain and discomfort, and anxiety and depression.  To measure the QoL in the TRACE study the following scores were used: the EQ-5D-5L questionnaire, the EQ-5D-5L VAS, the EQ-5D-5L index. |

## Overview of scores used in this study

| Revised Cardiac Risk Index (rCRI) Lee’s Revised Cardiac Risk Index: estimates patient’s risk in elective non-cardiac surgery patients of perioperative complications such as myocardial infarction, pulmonary edema, ventricular fibrillation or primary cardiac arrest, and complete heart block. [4]  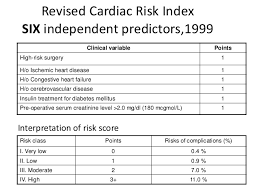Example: |
| --- |
| Global Surgical Recovery (GSR) index The GSR scores the extent to which patients consider themselves to be globally recovered from the surgery. This score is based on a single question. [5]   \| If 100% recovery is back to the usual health you had before you got sick and had surgery, what percent of recovery are you now?  …. % \| \| --- \|   Example: |
| Self-reported ADL recovery (ADL) The ADL scores whether the postoperative patient is able to fully perform daily activities. It consists of a single question in which patients report on a 2-point scale whether they were able to perform their daily activities fully or not.   \| Are you able to fully perform your daily activities again?   - I am still not able to fully perform my daily activities - I am able to fully perform my daily activities \| \| --- \|   Example: |

| Functional Recovery Index (FRI) The Functional Recovery Index (FRI) is derived from a questionnaire that assesses post-discharge functional recovery in the areas of pain, and social activity, lower limb activity and general physical activity. These are scored from 0 to 10, with 0 = no difficulty and 10 = extreme difficulty with the activity. The FRI is the sum of the scores per question. A lower score indicates better recovery, whereas a higher score indicates greater difficulty with recovery; the maximum score would be 140. [6]  Example:    In our study question number 7 was omitted because of the use of a separate pain questionnaire. The result of the grand score was thus the total score x 14 / number of answered questions which was max 13.  To correct for missing values on individual FRI items, we calculated adjusted sum scores: (sum score / number of valid answers) * 14 for the grand score, and (sum score / number of valid answers) * 7, 4 or 3 for the domains social, lower limb, and physical activity respectively. |
| --- |
| EQ-5D-5L Refers to either the EQ-5D-5L descriptive system or the EQ-5D-5L questionnaire.  Descriptive system. Descriptive system for health-related quality of life states in adults, consisting of five dimensions (Mobility, Self-care, Usual activities, Pain & discomfort, Anxiety & depression), each of which has five severity levels that are described by statements appropriate to that dimension.  Questionnaire. Standard layout for recording an adult person’s current self-reported health state. Consists of a standard format for respondents to record their health state according to the EQ-5D-5L descriptive system and the EQ-5D-5L VAS. [7]  Example:    Source: EQ-5D-5L user guide. [8] |
| EQ-5D-5L VAS Visual analogue scale of standard 20-cm layout, for recording an individual’s valuation of defined EQ-5D-5L profiles. The scale ranges from 100 (‘the best imaginable health state’) to 0 (‘the worst imaginable health state’). This is used to obtain a respondent’s stated preference values, not to record their own health state. [7]  Example:    Source: EQ-5D-5L user guide. |
| EQ-5D-5L (index) value The responses to the 5 EQ-5D-5L dimensions converted (with an index value calculator) into a single number, a summary index, called an index value.  The value attached to the EQ-5D-5L profile reflect, on average, people’s preferences about how good or bad the state is. Values are anchored at 1 (full health) and 0 (a state as bad as being dead) as required by their use in economic evaluation. Values less than 0 represent health states regarded as worse than a state that is as bad as being dead.  This value can be compared to value sets of a specific region/country. [7] |

# Supplementary Figures and Tables

## **Table S1.** Adverse events until one year after surgery

|  | Control | | Intervention | | aOR (95% CI)^#^ | p-value |
| --- | --- | --- | --- | --- | --- | --- |
|  | **Valid N** | **n (%)** | **Valid N** | **n (%)** |  |  |
| Mortality, 1-year** | 2456 | 133 (5.4) | 2676 | 156 (5.8) | 0.98 (0.74 – 1.31) | 0.897 |
| Readmission until 1 year | 1692 | 450 (26.6) | 1604 | 413 (25.7) | 0.91 (0.75– 1.10) | 0.305 |
| Reoperation until 1 year | 1696 | 347 (20.5) | 1613 | 292 (18.1) | 0.83 (0.68 – 1.02) | 0.074 |

** At 1 year, in total 221 out of 289 patients (76%) died with cancer (p = 0.30).

#adjusted OR for Group from Logistic mixed model with random intercept for hospital and fixed effects for cancer and renal failure at baseline

## **Table S2** Global Surgical Recovery Index

|  | Control* | | Intervention* | | P-value^#^ |
| --- | --- | --- | --- | --- | --- |
|  | **Valid N** | Median (IQR) | **Valid N** | Median (IQR) |  |
| **Recovered after surgery (%)** |  |  |  |  |  |
| Day 7 | 1840 | 60 (40-79) | 1882 | 60 (40-75) | 0.941 |
| Day 30 | 1854 | 75 (50-90) | 1856 | 75 (60-90) | 0.189 |
| Year 1 | 1747 | 90 (80-100) | 1679 | 95 (80-100) | 0.327 |
|  |  |  |  |  |  |
| **At baseline, expected recovery (%)** |  |  |  |  |  |
| Expected for Day 7 | 2288 | 60 (40-80) | 2124 | 50 (40-75) | 0.551 |
| Expected for Day 30 | 2287 | 85 (70-100) | 2142 | 90 (70-100) | 0.293 |

*Numbers depicted as: median (interquartile range) ^#^Independent samples Mann-Whitney U test

## **Figure S1.** Global Surgical Recovery and ADL performance

**
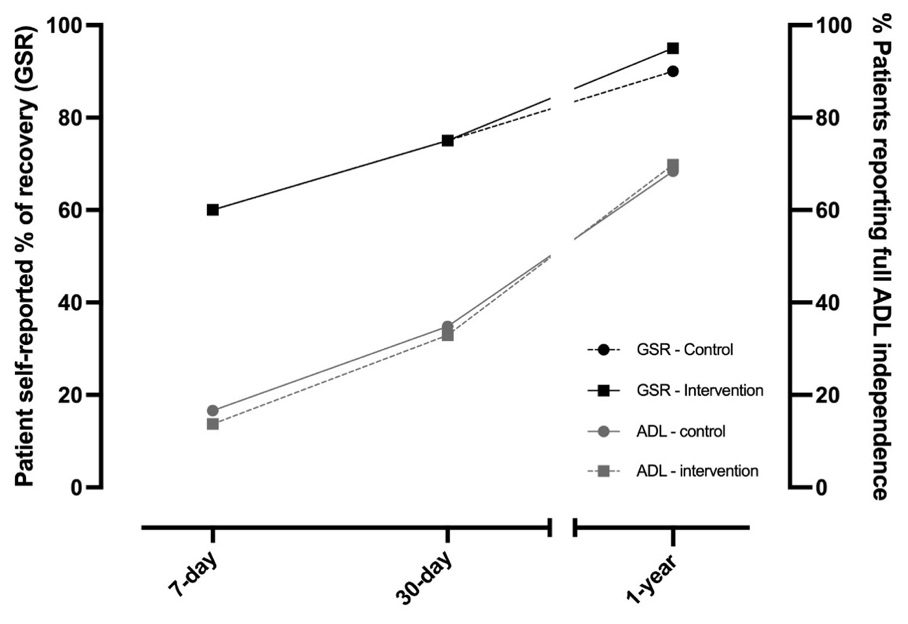
**

## **Table S3.** Ability to fully perform Activities of Daily Living (ADL)

|  | Control* | | Intervention* | | aOR (95% CI)^#^ | P-value |
| --- | --- | --- | --- | --- | --- | --- |
|  | **Valid N** | **n (%)** | **Valid N** | **n (%)** |  |  |
| Day 7 | 1827 | 303 (16.6) | 1851 | 253 (13.7) | 0.74 (0.60 – 0.92) | 0.007 |
| Day 30 | 1833 | 637 (34.8) | 1824 | 601 (32.9) | 0.91 (0.76 – 1.09) | 0.302 |
| Year 1 | 1650 | 1129 (68.4) | 1592 | 1112 (69.8) | 1.07 (0.89 – 1.30) | 0.466 |

*Numbers depicted as: N (%) #adjusted OR for Group from Logistic mixed model with random intercept for hospital and fixed effects for cancer and renal failure at baseline

## **Table S4a.** Functional Recovery Index

| **Table S4a. Functional Recovery Index (FRI) sum score - Overall** | | | | | |
| --- | --- | --- | --- | --- | --- |
|  |  | **Overall** | | | |
| **Group** | **Day** | **0** | **7** | **30** | **365** |
| Control | Valid N= | 2354 | 1858 | 1852 | 1685 |
|  | **Median** | 16 | 64 | 33 | 11 |
|  | IQR 25 | 0 | 29 | 9 | 0 |
|  | IQR 75 | 56 | 98 | 67 | 41 |
|  | **% Score ≥ 1** | 74.9% | 94.5% | 86.1% | 69.0% |
| Intervention | Valid N= | 2334 | 1914 | 1861 | 1612 |
|  | **Median** | 18 | 68 | 33 | 11 |
|  | IQR 25 | 1 | 32 | 9 | 0 |
|  | IQR 75 | 54 | 98 | 69 | 42 |
|  | **% Score ≥ 1** | 75.4% | 95.2% | 88.3% | 70.4% |
|  | **p-value*** | 0.882 | 0.299 | 0.684 | 0.649 |
| The values are presented as median, 25^th^, 75^th^ percentile.  Score ranges (adjusted for number of valid answers to the questions) overall and per domain, with higher scores meaning more difficulty: Overall 0 – 140, Social/pain 0 – 70, Lower limb 0-40, Physical 0 – 30.  A score ≥ 1 indicates having scored at least some functional difficulty in the given area.  * p-value from Independent-Samples Mann-Whitney U Test for sum score across study group | | | | | |

| **Table S4b. Functional Recovery Index (FRI) sum score – per domain** | | | | | | | | | | | | | |
| --- | --- | --- | --- | --- | --- | --- | --- | --- | --- | --- | --- | --- | --- |
|  |  | **Social/pain** | | | | **Lower limb** | | | | **Physical** | | | |
| **Group** | **Day** | **0** | **7** | **30** | **365** | **0** | **7** | **30** | **365** | **0** | **7** | **30** | **365** |
| Control | Valid N= | 2352 | 1845 | 1844 | 1667 | 2305 | 1638 | 1563 | 1377 | 2299 | 1590 | 1504 | 1307 |
|  | **Median** | 9 | 44 | 22 | 6 | 5 | 14 | 7 | 3 | 0 | 6 | 1 | 0 |
|  | IQR 25 | 0 | 20 | 6 | 0 | 0 | 5 | 0 | 0 | 0 | 1 | 0 | 0 |
|  | IQR 75 | 33 | 63 | 45 | 25 | 19 | 25 | 17 | 12 | 6 | 15 | 7 | 3 |
|  | **% Score ≥ 1** | 66.6% | 92.7% | 84.1% | 61.7% | 64.9% | 88.0% | 73.2% | 63.5% | 39.8% | 75.5% | 52.3% | 36.0% |
| Intervention | Valid N= | 2328 | 1885 | 1854 | 1590 | 2317 | 1577 | 1466 | 1403 | 2313 | 1508 | 1409 | 1320 |
|  | **Median** | 11 | 46 | 22 | 5 | 5 | 14 | 7 | 4 | 0 | 6 | 0 | 0 |
|  | IQR 25 | 0 | 21 | 6 | 0 | 0 | 5 | 1 | 0 | 0 | 0 | 0 | 0 |
|  | IQR 75 | 34 | 63 | 44 | 23 | 18 | 25 | 17 | 15 | 6 | 15 | 6 | 3 |
|  | **% Score ≥ 1** | 66.5% | 93.2% | 85.3% | 61.9% | 66.6% | 87.3% | 75.6% | 64.9% | 38.2% | 73.3% | 49.5% | 35.7% |
| The values are presented as median, 25^th^, 75^th^ percentile.  Score ranges (adjusted for number of valid answers to the questions) overall and per domain, with higher scores meaning more difficulty: Overall 0 – 140, Social/pain 0 – 70, Lower limb 0-40, Physical 0 – 30.  A score ≥ 1 indicates having scored at least some functional difficulty in the given area.  * p-value from Independent-Samples Mann-Whitney U Test for sum score across study group | | | | | | | | | | | | | |

## **Figure S2**. Functional Recovery Index

Median scores with Inter Quartile range. Range of score is 0 to 140, with higher scores meaning more difficulty.


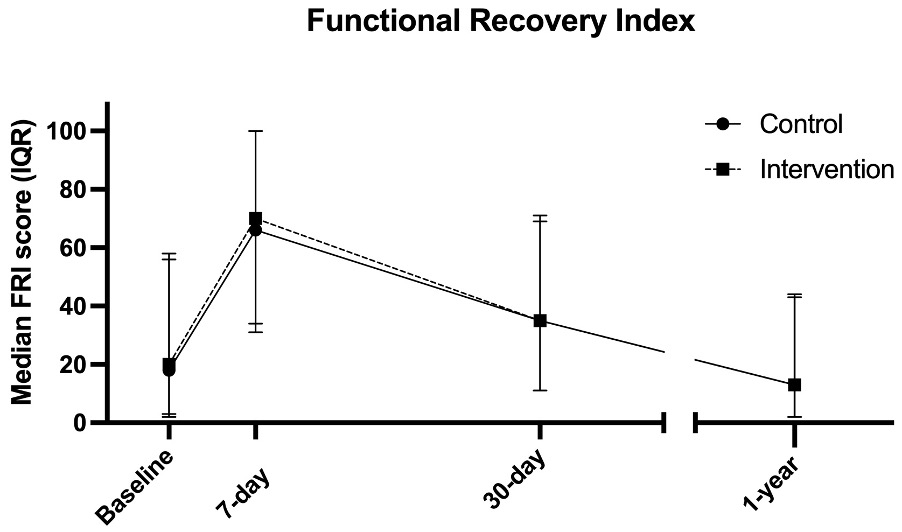


**Figure S3.** Functional Recovery Index overall and split per domain.

Proportion of patients reporting a score higher or equal than 1.

**
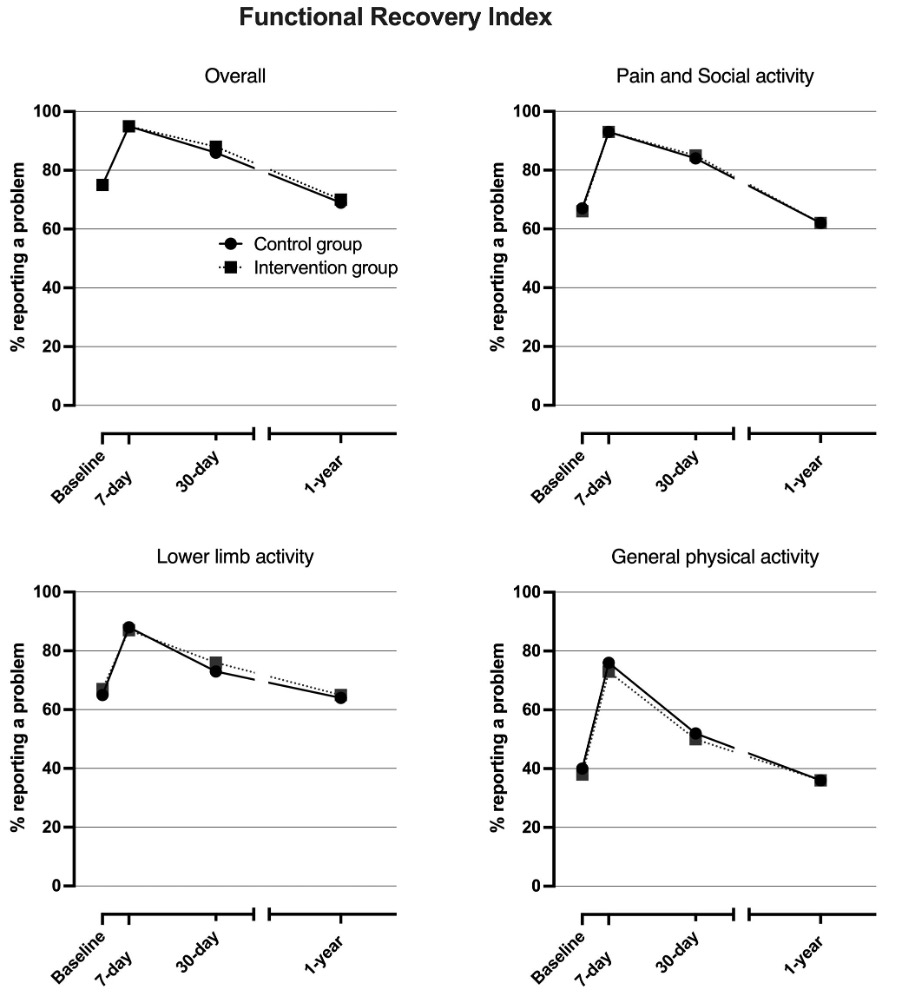
**

## Table S5: EQ-5D-5L index score

|  | Control | | Intervention | | p-value* |
| --- | --- | --- | --- | --- | --- |
|  | **Valid N** | **Median (IQR)** | **Valid N** | **Median (IQR)** |  |
| Baseline | 2342 | 0.82 (0.62-0.91) | 2306 | 0.82 (0.66-0.91) | 0.345 |
| Day 7 | 1794 | 0.74 (0.58-0.85) | 1770 | 0.74 (0.60-0.85) | 0.173 |
| Day 30 | 1764 | 0.81 (0.70-0.89) | 1743 | 0.81 (0.70-0.89) | 0.933 |
| Year 1 | 1614 | 0.86 (0.74-1.00) | 1566 | 0.88 (0.75-1.00) | 0.360 |

* p-value from Independent-Samples Mann-Whitney U Test for sum score across study group

## Table S6. Distribution of TRACE EQ-5D-5L dimension responses at baseline and at postoperative follow-up.

| **Table S6.** **Distribution of TRACE EQ-5D-5L dimension responses at baseline and at postoperative follow-up** | | | | | | | | | | | | | | | | | | | | | |
| --- | --- | --- | --- | --- | --- | --- | --- | --- | --- | --- | --- | --- | --- | --- | --- | --- | --- | --- | --- | --- | --- |
|  |  | **Mobility** | | | | **Self-care** | | | | **Usual activities** | | | | **Pain/discomfort** | | | | **Anxiety/depression** | | | |
| **Group** |  | **0** | **7** | **30** | **365** | **0** | **7** | **30** | **365** | **0** | **7** | **30** | **365** | **0** | **7** | **30** | **365** | **0** | **7** | **30** | **365** |
| Control | Valid N | 2380 | 1886 | 1903 | 1791 | 2377 | 1874 | 1888 | 1769 | 2362 | 1800 | 1786 | 1644 | 2374 | 1853 | 1856 | 1740 | 2361 | 1829 | 1823 | 1689 |
|  | 1 | 53.0 | 41.4 | 51.8 | 60.6 | 82.6 | 53.7 | 74.9 | 85.6 | 51.4 | 18.9 | 32.0 | 55.4 | 39.3 | 16.1 | 32.3 | 48.3 | 66.0 | 71.6 | 73.5 | 75.4 |
|  | 2 | 16.2 | 28.2 | 26.1 | 19.1 | 9.4 | 25.9 | 16.0 | 8.3 | 18.6 | 27.5 | 33.8 | 24.5 | 24.7 | 42.6 | 42.0 | 30.8 | 22.2 | 20.1 | 19.0 | 16.9 |
|  | 3 | 16.7 | 19.9 | 14.6 | 13.0 | 5.5 | 12.4 | 6.2 | 4.6 | 18.4 | 25.4 | 22.7 | 13.9 | 22.3 | 32.3 | 19.6 | 15.7 | 8.3 | 6.0 | 5.7 | 5.9 |
|  | 4 | 12.4 | 6.8 | 5.4 | 6.2 | 1.7 | 4.4 | 1.7 | 1.2 | 8.0 | 11.9 | 6.1 | 4.9 | 12.4 | 8.0 | 5.7 | 4.6 | 3.1 | 1.9 | 1.3 | 1.6 |
|  | 5 | 1.7 | 3.7 | 2.1 | 1.1 | 0.8 | 3.6 | 1.2 | 0.3 | 3.6 | 16.2 | 5.4 | 1.3 | 1.3 | 1.0 | 0.4 | 0.5 | 0.4 | 0.4 | 0.4 | 0.2 |
|  | **% score >1** | 47.0 | 58.6 | 48.2 | 39.4 | 17.4 | 46.3 | 25.1 | 14.4 | 48.6 | 81.1 | 68.0 | 44.6 | 60.7 | 83.9 | 67.7 | 51.7 | 34.0 | 28.4 | 26.5 | 24.6 |
| Intervention | Valid N | 2346 | 1941 | 1907 | 1724 | 2338 | 1923 | 1879 | 1694 | 2342 | 1797 | 1765 | 1605 | 2342 | 1897 | 1861 | 1676 | 2340 | 1858 | 1814 | 1632 |
|  | 1 | 53.1 | 40.1 | 50.6 | 58.5 | 83.4 | 56.0 | 75.9 | 86.5 | 50.8 | 17.6 | 29.3 | 55.4 | 39.8 | 16.0 | 30.9 | 49.2 | 67.9 | 73.5 | 75.0 | 75.6 |
|  | 2 | 16.7 | 29.7 | 26.6 | 20.8 | 9.3 | 25.9 | 15.5 | 8.7 | 21.9 | 29.3 | 36.7 | 24.4 | 25.5 | 45.9 | 44.3 | 29.8 | 22.7 | 18.6 | 18.2 | 18.7 |
|  | 3 | 15.2 | 20.2 | 16.2 | 14.6 | 4.7 | 11.9 | 6.1 | 3.4 | 16.7 | 26.4 | 22.7 | 15.5 | 21.9 | 30.0 | 19.8 | 16.1 | 6.5 | 6.1 | 5.3 | 4.5 |
|  | 4 | 13.6 | 7.3 | 4.6 | 5.7 | 1.4 | 3.5 | 1.3 | 0.8 | 7.1 | 11.1 | 6.7 | 3.3 | 11.3 | 7.0 | 4.8 | 4.7 | 2.4 | 1.5 | 1.3 | 1.0 |
|  | 5 | 1.4 | 2.7 | 2.0 | 0.5 | 1.2 | 2.8 | 1.2 | 0.6 | 3.5 | 15.6 | 4.6 | 1.4 | 1.5 | 1.1 | 0.2 | 0.2 | 0.6 | 0.3 | 0.2 | 0.2 |
|  | **% score >1** | 46.9 | 59.9 | 49.4 | 41.5 | 16.6 | 44.0 | 24.1 | 13.5 | 49.2 | 82.4 | 70.7 | 44.6 | 60.2 | 84.0 | 69.1 | 50.8 | 32.1 | 26.5 | 25.0 | 24.4 |
| Proportion of patients reporting answering options 1, 2, 3, 4, or 5, and proportion of patients reporting at least a problem (option 2-5) per dimension of the EQ-5D-5L. N = Total number of valid answers. | | | | | | | | | | | | | | | | | | | | | |

## Table S7. Results of post-hoc subgroup analyses – baseline characteristics

|  | **Intervention group – patients with recommendation(s)**  **N=582** | **Intervention group – patients without recommendation(s)**  **N=2118** |
| --- | --- | --- |
| Age (years) | 68 (61-74) | 67 (61-73) |
| Women | 315 (54.1%) | 985 (46.9%) |
| BMI (kg/m2) | 27 (24-30) | 27 (24-32) |
| Activity level (MET score <4) | 42 (7.3%) | 113 (5.4%) |
| **Functional status** |  |  |
| Independent | 517 (88.8%) | 1967 (92.9%) |
| Partially independent | 63 (10.8%) | 146 (6.9%) |
| Totally dependent | 2 (0.3%) | 5 (0.2%) |
| **ASA classification** |  |  |
| I | 48 (8.2%) | 184 (8.7%) |
| II | 356 (61.2%) | 1271 (60.0%) |
| III | 174 (29.9%) | 629 (29.7%) |
| IV | 4 (0.7%) | 33 (1.6%) |
| Unknown | 0 (0%) | 1 (0%) |
| **Comorbid disorders** |  |  |
| Active cancer | 285 (49.0%) | 868 (41.0%) |
| Hypertension | 280 (48.2%) | 907 (42.8%) |
| Cardiovascular disease | 167 (28.7%) | 539 (25.4%) |
| Cerebrovascular disease | 43 (7.4%) | 153 (7.2%) |
| Diabetes mellitus | 97 (16.7%) | 314 (14.8%) |
| Pulmonary disease | 63 (10.8%) | 220 (10.4%) |
| Renal failure | 60 (10.3%) | 291 (13.7%) |
| **Grade of surgery** |  |  |
| High risk surgery | 291 (50.0%) | 814 (38.4%) |

Data are median (IQR) or n (%). No formal statistical testing was applied.

## Table S8 Results of post-hoc subgroup analyses – outcome variables

|  | Intervention group – patients with recommendation(s)  N=582 | Intervention group – patients without recommendation(s)  N=2118 |
| --- | --- | --- |
| Mortality, 1-year | 43 (7.5%) | 113 (5.4%) |
| Readmission until 1 year | 118 (30.4%) | 295 (24.3%) |
| Reoperation until 1 year | 77 (19.8%) | 215 (17.6%) |
| Recovered after surgery (%) |  |  |
| Day 7 | 50 (30-70) | 60 (50-80) |
| Day 30 | 70 (50-80) | 80 (60-90) |
| Year 1 | 90 (70-100) | 95 (80-100) |
| Ability to fully perform ADL |  |  |
| Day 7 | 25 (5.9%) | 228 (16.0%) |
| Day 30 | 78 (18.8%) | 523 (37.1%) |
| Year 1 | 224 (57.6%) | 888 (73.8%) |
| FRI sum score |  |  |
| Day 7 | 26 (4-67) | 15 (0-49) |
| Day 30 | 87 (54-87) | 62 (28-91) |
| Year 1 | 23 (3-54) | 8 (0-36) |
| EQ-5D-5L index score |  |  |
| Day 7 | 0.66 (0.48-0.78) | 0.77 (0.63-0.85) |
| Day 30 | 0.77 (0.66-0.83) | 0.82 (0.72-0.91) |
| Year 1 | 0.81 (0.70-0.91) | 0.89 (0.78-1.00) |

Data are median (IQR) or n (%). No formal statistical testing was applied.

# References

1 Smit-Fun VM, de Korte-De Boer D, Posthuma LM, *et al.* TRACE (Routine posTsuRgical Anesthesia visit to improve patient outComE): A prospective, multicenter, stepped-wedge, cluster-randomized interventional study. *Trials* 2018;19(1):586.

2 Buhre WF, Boer C, de Korte-de Boer D, *et al.* Routine postsurgical anesthesia visit to improve 30-day morbidity and mortality: a multicenter, stepped-wedge cluster randomized interventional study (the TRACE Study). *Ann Surg.* 2023;277(3):375-380.

3 Hollmann MW, de Korte-De Boer D, Boer C, *et al.* The Routine posTsuRgical Anesthesia visit to improve patient outComE (TRACE) study: lessons learned. *Br J Anaesth.* 2021;127(4):e140-e142.

4 Lee TH, Marcantonio ER, Mangione CM, *et al.* Derivation and Prospective Validation of a Simple Index for Prediction of Cardiac Risk of Major Noncardiac Surgery. *Circulation.* 1999;100(10):1043-9.

5 M Kleinbeck Ã S v. Self-Reported At-Home Postoperative Recovery. *Res Nurse Health*. 2000;23(6):461-72.

6 Wong J, Tong D, de Silva Y, *et al.* Development of the Functional Recovery Index for Ambulatory Surgery and Anesthesia. *Anesthesiology.* 2009;110:596-602.

7 EuroQoL Terminology. https://euroqol.org/support/terminology/

8 EuroQoL. EQ-5D User Guide. https://euroqol.org/publications/user-guides/
